# Changes in Retinal and Choroidal Vascular Blood Flow after Oral Sildenafil: An Optical Coherence Tomography Angiography Study

**DOI:** 10.1155/2017/7174540

**Published:** 2017-10-09

**Authors:** David Berrones, Guillermo Salcedo-Villanueva, Virgilio Morales-Cantón, Raul Velez-Montoya

**Affiliations:** ^1^Ophthalmology Department, Asociación para Evitar la Ceguera en México IAP, Mexico City, Mexico; ^2^Retina Department, Asociación para Evitar la Ceguera en México IAP, Mexico City, Mexico

## Abstract

**Purpose:**

To describe changes in the retina and choroidal flow by optical coherence tomography angiography (OCT-A) after a single dose of oral sildenafil.

**Method:**

A case-control study. Patients in the study group received 50 mg of oral sildenafil. Patients in the control group received a sham pill. Retinal and choroidal images were obtained at baseline (before pill ingestion) and 1 hour after ingestion. Central macular and choroidal thickness, choroidal and outer retina flow, and the retinal and choroidal vascular density were compared using a Mann-Whitney *U* test.

**Results:**

Twenty eyes were enrolled into the study group and 10 eyes in the control group. There was a significant difference in central choroidal thickness and outer retina blood flow between groups after 1 hour of sildenafil ingestion (*p* < 0.01). There were no differences in central macular thickness, choroidal flow, and retinal vascular density among groups.

**Conclusions:**

A single dose of oral sildenafil increases choroidal thickness, probably due to sildenafil-induced vasodilation.

## 1. Introduction

Optical coherence tomography angiography (OCT-A) is a new noninvasive diagnostic tool that allows visualization of the retinal and choroidal circulation in real time. The study allows the estimation of the ocular vascular flow [1] and the theoretical quantification of the capillary density at different retinal anatomical zones. Although its role in patient care is still debatable, the study has shown promising results in demonstrating vascular changes occurring in common retinal pathologies like diabetic retinopathy, vascular occlusions, and choroidal neovascularization [2].

Sildenafil is a phosphodiesterase 5 and 6 (PDE-5, PDE-6) inhibitors that induce vasodilation. It is used in the treatment of pulmonary arterial hypertension and is currently the pharmacological first line treatment for erectile dysfunction. The drug achieves its biological effect by inducing the degradation of cyclic guanosine monophosphate (cGMP), enhancing the effect of nitric oxide and the inhibition of transmembrane influx of calcium and cell membrane hyperpolarization. The result is smooth muscle relaxation [3]. The recommended oral dose is 25–50 mg to 100 mg qd, three times per week. Doses above 100 mg can increase the risk of systemic adverse effects like headaches, nasal congestion, and flushing. Some ocular adverse effects are changes in color and light perception, blurred vision, conjunctival hyperemia, ocular pain, and photophobia [4]. Less common ocular adverse effects are nonarteritic ischemic optic neuropathy [5], reversible increase in intraocular pressure [6], idiopathic serous macular detachment, central serous chorioretinopathy [7, 8], and ERG disturbances [4, 9, 10].

A precise explanation of the ocular adverse effects is still unknown. However, it has been hypothesized that changes in the vascular flow of the retina and choroid could have an important role. Several studies have yielded mixed results. Those studies supporting this theory have shown that oral sildenafil can induce changes in choroidal thickness [11, 12], vascular flow [13–16], and alteration in retinal electroretinogram [17] and others that did not find changes in perimetry [18], flowmetry [19], and Doppler echography [20]. Therefore, the purpose of the current study is to assess vascular changes in the retinal and choroidal circulation, by taking advantage all the capabilities of the OCT-A, after the oral ingestion of sildenafil in healthy volunteers.

## 2. Method

Case and control, single center study. The study was conducted at *the Asociación para Evitar la Ceguera en México IAP*, Mexico City, Mexico and approved by the local Internal Review Board. The study was conducted according to the tenets of the Declaration of Helsinki and Good Clinical Practices guidelines. All sensitive data were managed according to the Mexican Federal Law for Protection of Personal Data in Possession of Individuals (NOM-024-SSA3-2010), which is the equivalent of the Health Insurance Portability and Accountability Act (HIPAA) rules of 1996. All participants signed an informed consent form before enrollment.

We included patients between 18 and 40 years of age, with no relevant medical history. Patients with history of a hypersensitivity reaction to phosphodiesterase inhibitors or history of past adverse effect with oral sildenafil were excluded. General demographic data and biometric data (weight and height) were recorded at baseline. All participants were stratified randomized into two groups with a proportion of 2 : 1. Patients assigned to the study group received 50 mg of oral sildenafil (Viagra®, Pfizer, New York, NY) and patients assigned to the control group received a sham pill the same size, color, and form.

Retinal and choroidal images were captured at baseline and 1 hours after the pill ingestion. Central macular thickness (CMT) and central choroidal thickness (CCT) were measured with optical coherent tomography (OCT) and enhanced depth imaging-OCT (Spectralis HRA-OCT; Heidelberg Engineering, Heidelberg, Germany). Central choroidal thickness was measured using the measuring tool built in the OCT software. The measurements were done in three locations: subfoveal, 1000 *μ*m temporal to the fovea, and 1000 *μ*m nasal to the fovea. All three measurements were averaged into a single central choroidal thickness value per patient. All measurements were done in triplicates. Choroidal flow (CF), outer retina flow (ORF), retinal vascular density (RVD), and choroidal vascular density (CVD) were measured with OCT-A (AngioVue OCT-A system, Optovue Inc., Fremont, CA).

All OCT-A images were taken using the default setting of the system (70,000 A scans per second, ≈5 *μ*m on optical axial resolution, and ≈15 *μ*m on optical transversal resolution). Segmentation of the images was done with the automatic segmentation tool build in the software. All values were derived directly from the calculation made by the system and shown on the screen.

Statistical analysis was done using an Excel spreadsheet (Excel 2010; Microsoft Corp., Redmond, WA) with and XLSTAT application v18.06 (Addinsoft, New York, NY). Continuous variables were compared using a Mann-Whitney *U* test with an alpha value of 0.05 for statistical significance.

## 3. Results

We enrolled 20 eyes into the study group (sildenafil 50 mg) and 10 eyes into the control group (sham). In the study group, the mean age was 28.1 years, the mean weight was 73.6 kg, and the mean height was 1.76 meters. In the control group, the mean age was 29.2 years, the mean weight was 70.4 kg, and the mean height was 1.75 meters.

Results from the study and control group are summarized in Table 1. In the study group, CCT increased significantly after 1 hour of sildenafil ingestion (*p* < 0.01). The rest of the assessed variables in the study group did not have a significant change after sildenafil. In the control group, none of the assessed variables had a significant change after sham.

Intergroup comparison between study and control group are summarized in Table 2. The analysis showed that 1 hour after pills ingestion, the outer retinal flow increased significantly in the study group (*p* < 0.01). The rest of the assessed variables between groups did not have a significant difference between them.

## 4. Discussion

Optical coherence tomography angiography is a novel diagnostic tool that can potentially impact ocular medical therapies in the future. It has been tried as an early diagnostic and assessment tool for age-related macular degeneration (AMD) [21–24], as a research tool in order to improve the current understanding of ischemic diseases that affects the different layers of the retinal microcirculation (diabetic retinopathy) [25, 26], cotton wool spots, acute macular neuroretinopathy, paracentral acute middle maculopathy, and macular telangiectasia type 2 among others [27–29].

The role of vascular flow changes in choroidal diseases is unknown. Evidence from peer-reviewed manuscripts has found a relationship between flow changes among different retinal layers and choroid with the pathogenesis of some of the most common chorioretinal diseases [30–33]. Changes in choroidal thickness (reduction) and in the density of superficial and deep retinal vessels have been associated with choroidal neovascularization [34–36]. A reduction in the total retinal and choroidal flow has been observed in patients with macular edema [37, 38], and finally, an increase in the total choroidal flow has been observed in patients with central serous retinopathy [39]. Since most of the ocular side effects of oral sildenafil are thought to be secondary to an idiosyncratic dilation of the retinal and choroidal vessels, the potential impact of this pharmacologic effect on the total vascular flow should be assessed. A major understanding of the mechanisms associated with this phenomenon could aid us in preventing such adverse effects as well as improving our current understanding of the relationship of vascular flow and others chorioretinal diseases like central serous retinopathy.

The results of the current study are similar to previous reports reporting a significant increase in choroidal thickness measured by EDI-OCT. However, OCT-A results failed to demonstrate a significant change in choroidal blood flow. Outer retinal blood flow increased after sildenafil, but the capillary density of the retina remained unchanged. Nevertheless, our results may suggest that despite a local pharmacological effect secondary to sildenafil, the autoregulation mechanisms of the retinal and choroidal vasculature may prevent significant changes in vascular hemodynamics, at least in the short term, ensuring a constant blood flow and preventing blood stagnation and ischemic changes in healthy patients.

The current study has several limitations that we would like to address. First, an error type 2 is highly likely due to the small sample. The high variability in biometric values could affect the distribution volume of the drug among patients. Therefore, our patients could have responded differently to a fixed dose. There is a possibility that an ocular vascular effect could have been demonstrated if we had adjusted the dose according to weight and height. Finally, all our patients were first-time users of sildenafil. Most of the ocular adverse effects of the drug occur in chronic users. There is a possibility that ocular changes due to sildenafil are due to the continued exposition to the drug or to the association of a preexisting ocular condition with exposition to the drug. Finally, it is important to note that the AngioVue OCT-A default setting is programmed to measure the choroidal flow solely at the level of the choriocapillaris, while EDI software is designed to measure the whole choroidal thickness. This is an important difference to consider since this might be the reason why there was no change observed in choroidal flow measurements after sildenafil intake.

In summary, our study found an increase in choroidal thickness and in the outer retinal flow that suggests a vascular effect of sildenafil in the eye. No measurable data was found regarding the change in choroidal flow, despite the increase in choroidal thickness. The results may suggest that even with a local pharmacological effect of sildenafil, the vascular autoregulation mechanisms of the retina and choroid may prevent a significant change in vascular hemodynamics, at least in the short term, ensuring a constant blood flow and preventing blood stagnation and ischemia. Further studies with longer follow-up are needed in order to assess the role of the chronic use of sildenafil on retinal vascular hemodynamics in healthy and diseased patients.

## Figures and Tables

**Table 1:** Change in OCT before and after sildenafil/sham. Comparison of variables before and after the ingestion of the pill for both groups. CMT: central macular thickness; CCT: central choroidal thickness; CF: choroidal flow; ORF: outer retinal flow; RVD: retinal vascular density; CVD: choroidal vascular density. ^∗^*p* < 0.01.

	Before	After
*Sildenafil group*		
CMT	280.4 ± 23.69 *μ*m	279.85 ± 22.80 *μ*m
CCT	354.45 ± 67.17 *μ*m	429.30 ± 82.87 *μ*m^∗^
CF	1.95 ± 0.04 mm^2^	1.87 ± 0.22 mm^2^
ORF	1.21 ± 0.20 mm^2^	1.31 ± 0.27 mm^2^
RVD	52.6 ± 3.50%	53.6 ± 3.21%
CVD	66.6 ± 1.48%	67.28 ± 1.02%
*Sham group*		
CMT	266.60 ± 19.28 *μ*m	266.20 ± 19.02 *μ*m
CCT	397.10 ± 72.81 *μ*m	396.50 ± 68.48 *μ*m
CF	1.96 ± 0.05 mm^2^	1.95 ± 0.05 mm^2^
ORF	1.06 ± 0.23 mm^2^	1.03 ± 0.23 mm^2^
RVD	53.9 ± 2.16%	53.3 ± 3.45%
CVD	66.98 ± 1.06%	66.90 ± 1.50%

**Table 2:** Results after pill administration for both groups. Comparison of the variables for the study and control group 1 hour after the ingestion of sildenafil/sham. CMT: central macular thickness; CCT: central choroidal thickness; CF: choroidal flow; ORF: outer retinal flow; RVD: retinal vascular density; CVD: choroidal vascular density. ^∗^*p* < 0.01.

	Posttreatment sildenafil group	Posttreatment sham group
CMT	279.85 ± 22.80 *μ*m	266.20 ± 19.02 *μ*m
CCT	429.30 ± 82.87 *μ*m	396.50 ± 68.48 *μ*m
CF	1.87 ± 0.22 mm^2^	1.95 ± 0.05 mm^2^
ORF	1.31 ± 0.27 mm^2^	1.03 ± 0.23 mm^2^^∗^
RVD	53.6 ± 3.21%	53.3 ± 3.45%
CVD	67.28 ± 1.02%	66.90 ± 1.50%
